# Pulmonary Thromboembolism and Infarction Mimicking COVID-19 Symptoms; Report of three Cases

**Published:** 2020-11-02

**Authors:** Rama Bozorgmehr, Mehdi Pishgahi, Zohreh Tajabadi, Mohammad Aryafar

**Affiliations:** 1 Clinical Research Development Unit, Shohadaye Tajrish Hospital, Shahid Beheshti University of Medical Sciences, Tehran, Iran.; 2 Cardiology Research Center, Shohadaye Tajrish Hospital, Shahid Beheshti University of Medical Sciences, Tehran, Iran.; 3 Student Research Committee, School of Medicine, Shahid Beheshti University of Medical Sciences, Tehran, Iran.; 4 4. Department of Anesthesiology, Tehran Medical Sciences Branch, Islamic Azad University, Tehran, Iran.

**Keywords:** Pulmonary embolism, pulmonary infarction, COVID-19, venous thrombosis, signs and symptoms, respiratory

## Abstract

Since the novel coronavirus emerged in late December, 2019 in Wuhan, China, millions of people have been infected and thousands of patients have died. Fever and dyspnea are the most common symptoms of infection with SARS-CoV-2. However, these symptoms are neither specific nor diagnostic for COVID-19. Symptom overlap between COVID-19 and some other conditions may lead other diseases to be missed and underdiagnosed. Just like COVID-19, pulmonary thromboembolism (PTE) and pulmonary infarction may present with fever and respiratory symptoms. Since COVID-19 emerged and spread worldwide, many clinicians are focused on diagnosis and treatment of this novel viral infection. Hence, other diseases presenting with the same symptoms as COVID-19 may remain underdiagnosed. Here, we report three cases of PTE and pulmonary infarction presenting with fever and respiratory symptoms mimicking COVID-19.

## Introduction

SARS-CoV-2 is a single-strand RNA virus, which belongs to Coronaviridae family (1). Since the novel coronavirus emerged in late December, 2019 in Wuhan, China, millions of people have been infected and thousands of patients have died. Nowadays, COVID-19 pandemic is a major concern for both people and health-care systems. Fever and dyspnea are the most common symptoms of infection with SARS-CoV-2 (2). However, these symptoms are neither specific nor diagnostic for COVID-19. Many other diseases initially present with fever and respiratory symptoms. Symptom overlap between COVID-19 and some other conditions may result in other diseases being missed and underdiagnosed. Underdiagnosis of the disease may increase the risk of morbidity and mortality due to misdiagnosis, mistreatment, or delay in treatment initiation.

Like COVID-19, pulmonary thromboembolism (PTE) may present with fever and respiratory symptoms such as dyspnea, tachypnea and hemoptysis (1). PTE occurs among one third of patients with deep vein thrombosis (DVT) and is associated with increased risk of morbidity and mortality (3). Furthermore, recent researches have demonstrated that COVID-19 is associated with coagulopathy and increases the risk of DVT and PTE (4). Due to overlap of symptoms and findings between these conditions, diagnosis of these diseases is challenging. 

During COVID-19 pandemic, pulmonary thromboembolism, pulmonary infarction, and other diseases presenting with fever and respiratory symptoms should also be considered to prevent misdiagnosis of these diseases. In this series, pulmonary thromboembolism and associated pulmonary infarction mimicking the symptoms and findings of COVID-19 is reported in a young post-partum woman with history of thrombosis, an old woman with a history of recent trauma, and a young man without any history of underlying medical diseases.

## Case presentations:

***Case 1***

A 29-year-old postpartum woman from Qom city, where the first COVID-19 case in Iran was observed, presented to our emergency department with a history of dyspnea, tachypnea, and pleuritic chest pain. At the time of admission, an O_2_ saturation of 92%, heart rate of 108 bpm, respiratory rate of 28/minutes and oral temperature of 38 ^0^C were noted. Her blood pressure was within the normal range. Physical examination revealed edema, tenderness, and warmth of the right leg. The left leg was normal in physical examination. Electrocardiogram was normal.

At the time of admission, laboratory tests revealed normal white blood cell count (6.2×10^3^/mm^3^), platelet count (226×10^3^/mm^3^), blood urea nitrogen (14 mg/dl) and creatinine (0.8 mg/dl). In blood examination, hemoglobin =10.1 gr/dl, prothrombin time=16 seconds, international normalized ratio (INR) =1.4, and erythrocyte sedimentation rate (ESR) = 50 mm/hour were noted. Liver function tests were normal. Other laboratory parameters were in normal ranges. Due to dyspnea and fever, spiral chest computed tomography (CT) scan was done to assess COVID-19, which had some bilateral and sub-pleural patchy ground glass infiltration. The radiologist report was consistent with COVID-19. But the patient had a history of left leg pain and edema from seven days ago and because of that we considered pulmonary thromboembolism and pulmonary infarction.

The patient had a previous history of left transverse and sigmoid sinus thrombosis, right cavernous sinus thrombosis, and several episodes of seizure six years ago. The patient was on heparin treatment for six years but had received no treatment during pregnancy. 

**Figure 1:**
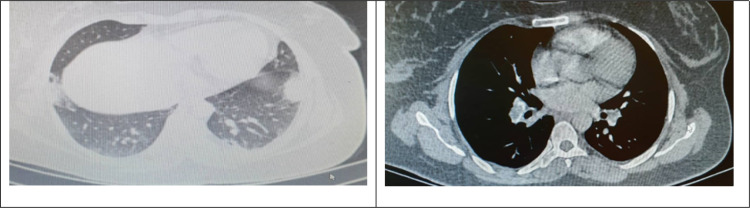
Central filling defect in right inter-lobar artery and left lower segmental artery with infarction of both peripheral superior segments of right lower lobe and left lower lobe

Since the symptoms and CT scan findings were consistent with both COVID-19 and pulmonary infarction, the reverse transcriptase polymerase chain reaction (RT-PCR) test and a multi-detector CT angiography of lungs were performed. The RT-PCR test showed negative results. CT angiography demonstrated evidences of central filling defect in left lower artery and right inter-lobar artery extending to segmental branches (Figure 1). Furthermore, bilateral wedge-shaped consolidation of peripheral superior segment was found. Findings of CT angiography were consistent with pulmonary thromboembolism (PTE) and pulmonary infarction. 

She also underwent color-flow Doppler sonography, which indicated abnormal venous flow in right common femoral, superficial femoral, popliteal, and external iliac veins indicating acute deep vein thrombosis (DVT) in these veins. During compression ultrasound, the veins were unable to compress. Venous blood flow was normal through inferior vena cava. Echocardiography showed normal ventricular size and function. No evidence of pulmonary thromboembolism was detected on echocardiography.

Therefore, due to previous history of thrombophilia and concurrent DVT, pulmonary thromboembolism leading to pulmonary infarction was diagnosed and anticoagulant therapy was started. 

Several days after admission, she developed fever and chills, and dyspnea worsened. Due to a high suspicion of COVID-19, both RT-PCR test and IgM and IgG antibody tests for COVID-19 were performed, which showed negative results and COVID-19 was ruled out. Since post-partum women are at increased risk of infections and also pulmonary infarction is associated with a greater risk of infections, pneumonia was considered in this patient. Therefore, intravenous antibiotic therapy was initiated. Subsequently, sputum smear and culture for bacterial infections were performed, which showed negative results. Furthermore, all thrombophilic evaluations were negative.

During hospitalization, leg pain and edema improved gradually. Fever and chills resolved. Patient’s clinical condition significantly improved and she was finally discharged from the hospital. 

***Case 2***

An 80-year-old woman from Tehran presented to our emergency department with a history of one week of dyspnea, productive cough, and left-sided pleuritic chest pain. The patient had a history of asthma and was receiving salbutamol. She had a history of falling down resulting in a blunt chest trauma about three weeks ago. At the time of admission, an O_2_ saturation of 93% and a respiratory rate of 24/minutes was noted. Heart rate, blood pressure, and oral temperature were within normal ranges. On physical examination, inspiratory basal crackles in left lung were noted. The right lung was clear on auscultation. An electrocardiography was performed, which was normal.

At the time of admission, laboratory tests revealed normal white blood cell count (5.9×10^3^/mm^3^), platelet count (314×10^3^/mm^3^), ESR (6 mm/hour), c-reactive protein (CRP) (5.6 mg/dl), and creatinine (1.32 mg/dl). In blood examination, blood urea nitrogen = 30 mg/dl, hemoglobin = 11.3 gr/dl, Calcium = 11.15 mg/dl, and phosphorus = 5.5 mg/dl were noted. Venous blood gas analysis was normal for pH (7.39), PCO_2_ (40 mmHg) and HCO_3_ (23.9 mEq/L). Other laboratory parameters were within normal ranges.

**Figure 2:**
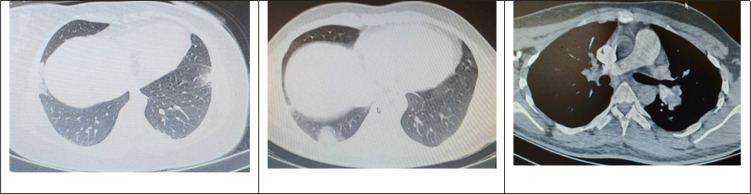
Filling defect in left main pulmonary artery and right lower lobe with bilateral sub-pleural infarction

Due to respiratory symptoms, a spiral chest CT scan was performed on admission, which showed ground glass opacities of sub-pleural regions in left lung. CT imaging revealed multiple consolidations and atelectasis in right lung. The radiologist report was consistent with COVID-19. However, since symptoms and imaging findings were consistent with both COVID-19 and pulmonary infarction, the patient underwent RT-PCR test and CT angiography. The RT-PCR test revealed negative results. CT angiography of lungs showed evidence of filling defect in left pulmonary artery extending to segmental branches, which was consistent with pulmonary infarction due to pulmonary embolism (figure 2). CT angiography revealed no abnormal findings in right lung. Subsequently, the patient underwent color-flow Doppler sonography of lower extremities to find evidence of any vein thrombosis. However, no findings of acute DVT were reported. Since the diagnosis of PTE and pulmonary infarction was confirmed, anticoagulant therapy was started. 

During hospitalization, the levels of hemoglobin gradually decreased to 10.7 gr/dl, and ESR and CRP increased to 24 and 33.2 mg/dl, respectively. 

During hospitalization, dyspnea and pleuritic chest pain improved and productive cough was resolved gradually. After six days, the patient was discharged without any complaints or complications.

***Case 3***

A 23-year-old man presented to our emergency department with a history of progressive dyspnea and dry cough from 10 days ago. He also complained of right-sided chest pain that exacerbated by lying flat, which had started 3 days ago. Fever and sore throat also started one day ago. He had a history of exposure to COVID-positive patients during the last two weeks. The patient denied any history of underlying medical diseases. He also denied any history of smoking, alcohol, or drug use. At the time of admission, a heart rate of 125 beat/minute and blood pressure of 100/75 mmHg were noted. O_2_ saturation was 91% in room air, which increased to 97% with mask. Respiratory rate and oral temperature were normal. Physical examinations did not reveal any abnormalities. The heart and lungs were clear to auscultation.

On admission, blood examinations revealed white blood cell count (WBC) =7500×10^3^ /mm^3^; hemoglobin =14.9 gr/dl; Platelet = 196000; CRP=10 mg/dl and ESR = 7 mm/hour, all of which were within normal ranges. Biochemical tests showed creatinine = 0.9 mg/dl; blood urea nitrogen (BUN) = 13 mg/dl; Na = 140 meq/L; K = 3.5 meq/L; creatine phosphokinase (CPK) = 162 IU/L; creatine kinase-MB (CKmb) = 21 IU/L, and lactate dehydrogenase (LDH) = 381 U/L. Venous blood gas analysis demonstrated a pH of 7.35, PCO_2_ of 51.9 mmHg, and HCO_3_ of 27.9 mEq/L, which were indicative of respiratory acidosis.

**Figure 3:**
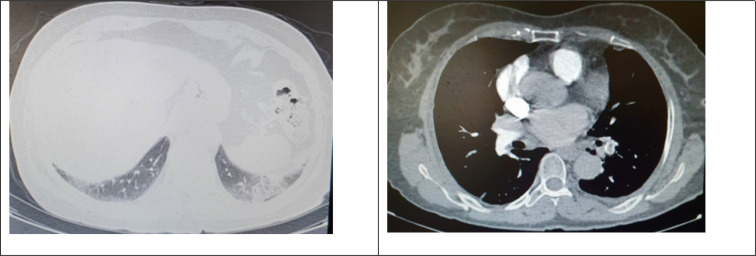
Central filling defect in main pulmonary branch of left lower lobe with infarction in posterior-basal segment of left lower lobe on pulmonary angiography

Due to fever, respiratory symptoms, and recent history of exposure to COVID-positive patients, initially, COVID-19 was suspected. The patient underwent spiral CT scan of lungs, which revealed bilateral and sub-pleural ground glass opacities. Since symptoms and imaging findings were consistent with both COVID-19 and pulmonary infarction, the patient underwent RT-PCR test and CT angiography. The result of RT-PCR test for COVID-19 was negative. CT angiography of lungs showed massive PTE in both right and left pulmonary arteries (figure 3). Furthermore, peripheral patchy lesions without enhancement were seen, which were indicative of pulmonary infarction in both lungs. Therefore, infection with SARS-CoV-2 was ruled out and the diagnosis of pulmonary infarction due to PTE was confirmed for the patient by performing echocardiography and color-flow Doppler sonography of the abdomen and lower limbs. Echocardiography revealed a Large PTE and a severely dilated right ventricle. However, color-flow Doppler sonography showed no evidence of acute DVT. Furthermore, mesenteric veins had normal blood flow and peak systolic velocity.

Since the diagnosis of PTE and pulmonary infarction was confirmed using imaging, fibrinolytic and anticoagulant therapy were started. Prothrombine time (PT), partial thromboplastin time (PTT), and INR were closely monitored. During hospitalization, dyspnea, productive cough, and chest pain resolved. The fever improved gradually. On the last day of hospitalization, blood tests revealed WBC=5100/mm^3^; hemoglobin = 14.9 gr/dl; platelet count = 233000/mm^3^; PT = 13 seconds; INR = 1 and PTT = 32 seconds, which were all within normal ranges. The patient was discharged on the sixth day without any complications.

## Discussion

COVID-19 emerged in winter, 2019 in Wuhan province, China and rapidly spread all over the world. In March 2020, world health organization declared COVID-19 as a pandemic (5). To date more than 20,000,000 people have been infected around the world. Infection with this novel coronavirus is associated with a considerable rate of morbidity and mortality. COVID-19 usually presents with fever and respiratory symptoms including dyspnea, tachypnea, cough, and hemoptysis. These symptoms are neither specific nor diagnostic for COVID-19. Many other infectious or respiratory disorders also present with constitutional and respiratory symptoms. Hence, during COVID-19 pandemic, for the patients presenting with fever and respiratory symptoms, other diseases should also be considered to prevent misdiagnosis of the underlying disease.

Pulmonary thromboembolism and pulmonary infarction are among the respiratory disease, which usually present with respiratory symptoms such as dyspnea, chest pain, and hemoptysis.

Recent researches have indicated that COVID-19 increases the risk of thrombosis development. A recent study carried out by Pishgahi et al., reported massive thromboembolism development among three ICU admitted patients with COVID-19 (6). Patients with COVID-19 usually suffer from hypoxia and sepsis, which are among the major causes of thrombosis formation (4). Furthermore, the novel coronavirus can infect cells via ACE2 receptor, which results in endothelial damage (6). However, the exact mechanism is unknown. COVID-19 can induce inflammation, platelet activation, endothelial dysfunction, and stasis, leading to thrombotic events such as DVT and PTE (7). Thrombotic events are associated with increased risk of death among patients infected with SARS-CoV-2 virus (4).

Deep vein thrombosis accounts for two-thirds of venous thromboembolism cases, occurring in 1 out of 1000 individuals each year (3). Venous stasis, vascular injury, and hypercoagulability, known as “Virchow’s Triad”, are the major factors leading to thrombosis formation (3). Pregnancy, prolonged immobility, and previous history of thrombosis are associated with an increased risk of DVT. Our first patient had the history of recent pregnancy, previous hypercoagulability, and cerebral vein thrombosis associated with increased risk of thrombotic events. Patients with DVT usually present with leg pain, warmth sensation, swelling and leg ulcers (3). Also patients with DVT may be asymptomatic (7). Because of safety, cost-effectiveness, and reliability, ultrasound imaging is the first modality used for the diagnosis of DVT (3). Color-flow Doppler sonography is also used for better evaluation of thrombosis. The reported patients underwent color-flow Doppler sonography. The first patient had no normal venous flow in right common femoral, superficial femoral, popliteal, and external iliac veins, indicating acute DVT in these veins. These findings were consistent with DVT. However, other patients showed no evidence of vein thrombosis in color-flow Doppler sonography. 

Sometimes DVT may progress to PTE. PTE is among the major manifestations of venous thromboembolism. Pulmonary thromboembolism can present with a wide range of non-specific symptoms, which makes the diagnosis of the disease difficult (8). Studies showed that patients with COVID-19 are at an increased risk of PTE development. Ohana et al., reported that 30% of patients with COVID-19 developed PTE, which was greater than PTE rate among ill patients without SARS-CoV-2 infection (9). 

PTE can lead to pulmonary infarction, which is a rare complication of pulmonary embolism (10). Pulmonary infarction does not occur frequently due to robust oxygenation supports and vascular supplies of lungs from pulmonary circulation, bronchial circulation, and airways (11). Risk factors for pulmonary infarction are the same as DVT and PTE (12). Pulmonary infarction may present with chest pain, bleeding, and hemoptysis. However, pleuritic chest pain and sudden dyspnea are the most common symptoms of pulmonary infarction (11). These three patients all presented with dyspnea and chest pain. A retrospective study carried out by Chengsupanimit et al., reported that six-month survival of patients who develop pulmonary infarction was 88%. Also researchers found that 55% of patients with pulmonary infarction had a history of DVT (10). 

Concurrent peripheral consolidation and central clearance, mixed peripheral consolidation with ground glass opacity (GGO), and peripheral GGO are among the most common CT scan findings in patients who develop pulmonary infarction (10). On the other hand, GGO, mixed GGO, crazy-paving patterns, peripheral and subpleural consolidations, and bilateral involvement of both lower lungs are among the typical findings of CT scan among patients with COVID-19 (13). Therefore, in the presence of these findings, both COVID-19 and pulmonary infarction should be considered. The reported patients underwent CT scan of lungs, which revealed multiple consolidations and ground glass opacities. CT scan findings were consistent with both COVID-19 and pulmonary infarction. COVID-19 was considered first. However, reverse transcriptase polymerase chain reaction for COVID-19 showed negative results and other diseases were considered.

During COVID-19 pandemic, some of the diseases may remain underdiagnosed. COVID-19 usually presents with constitutional symptoms such as fever, chills, myalgia, fatigue, and respiratory symptoms including dyspnea, cough, and tachypnea, and in some cases hemoptysis. Many other clinical situations such as malignancies, anemia, metabolic disorders, and infections may also initiate with constitutional symptoms. Furthermore, respiratory disorders such as upper respiratory tract infections, pneumonias, pulmonary embolism, and pulmonary infarction can present with respiratory symptoms. Since COVID-19 emerged and spread worldwide, many clinicians are focused on diagnosis and treatment of this novel viral infection. Hence, other diseases presenting with the same symptoms as COVID-19 may remain underdiagnosed. In the reported cases, PTE and pulmonary infarction presented with fever and respiratory symptoms, mimicking COVID-19. Since there was high suspicion for COVID-19, diagnostic tests were performed, which showed negative results. After COVID-19 was ruled out, the diagnosis of underlying pulmonary condition was considered. CT angiography of lung confirmed the diagnosis of PTE associated with pulmonary infarction.

When the diagnosis of pulmonary infarction due to PTE was confirmed, anticoagulant therapy was started. Studies showed that anticoagulant therapy decreases the risk of thrombosis recurrence (3). Also, recent researches demonstrated that thromboprophylaxis and anticoagulant therapy lower the risk of mortality among patients with severe COVID-19 (4). During hospitalization, patients’ clinical conditions improved significantly. At the time of writing this manuscript, these patients are still symptom-free.

## Conclusion:

Since COVID-19 emerged and spread worldwide, many health-care workers have focused on diagnosis and treatment of this viral infection. Similarities between symptoms of COVID-19 and many other diseases makes the diagnosis of the underlying disease harder during COVID-19 pandemic. Under-diagnosis of the diseases leads to delay in diagnosis and treatment initiation, which may increase the risk of patient morbidity and mortality. Therefore, it is important to consider other underlying diseases presenting with constitutional and respiratory symptoms during COVID-19 pandemic.
